# Local Injection Therapy to Relieve Residual Pain in Osteoporotic Vertebral Fracture Patients With Fascia Injury Treated by Kyphoplasty

**DOI:** 10.1155/prm/8470601

**Published:** 2026-11-15

**Authors:** Zicheng Deng, Luyang Li, Tao Feng, Yufan Ying, Dawei Song, Shengyang Jin, Qi Yan, Jun Zou, Jinning Wang, Junjie Niu

**Affiliations:** ^1^ Department of Orthopaedic Surgery The First Affiliated Hospital of Soochow University NO. 899, Pinghai Road Suzhou 215006 China sdfyy.cn

**Keywords:** local injection therapy, osteoporotic vertebral fracture, percutaneous kyphoplasty, residual back pain, thoracolumbar fascia injury

## Abstract

**Background:**

Thoracolumbar fascia injury (FI) which has been demonstrated to be related to residual pain after kyphoplasty has received increasing attention. However, there is currently no definite treatment for FI in osteoporotic vertebral fracture (OVF) patients. This study aimed to evaluate the efficacy of percutaneous kyphoplasty (PKP) combined with local injection therapy for the treatment of OVF patients with FI.

**Methods:**

Seventy‐one OVF patients with FI were included in this study and were divided into two groups (Group 1, kyphoplasty without local injection therapy; Group 2, with local injection therapy). The demographics of patients between the two groups were compared. Then the clinical efficacy was compared between the two groups preoperatively and at different follow‐up time points. The patients in Group 2 were further classified into two groups based on the severity of FI and the clinical efficacy was evaluated between these two groups.

**Results:**

The operative time was significantly longer in Group 2 than in Group 1. In Group 2, the visual analogue scale (VAS) and Oswestry disability index (ODI) scores were significantly lower one day and 1 month postoperatively compared to the values in Group 1. The satisfaction survey also presented that more patients were satisfied with the surgery one day postoperatively in Group 2. Further comparison between the patients with severe or nonsevere FI showed that there were no statistically significant differences in VAS and ODI scores at each follow‐up time point.

**Conclusions:**

Local injection therapy combined with PKP is a safe and effective strategy for improving early postoperative recovery and reducing early residual pain in OVF patients with FI.

## 1. Introduction

Osteoporotic vertebral fractures (OVFs) are common in elderly patients, which decrease patients’ quality of life and result in a great economic burden to society [1]. With the aging of the population, the incidence of OVFs is increasing annually. Vertebral augmentation, known as percutaneous kyphoplasty (PKP) or vertebroplasty (PVP), is often used to treat OVFs, which can stabilize the fracture, effectively restore vertebral height, relieve pain, and improve patients’ mobility [2–4]. However, postoperative residual pain which is defined as visual analogue scale (VAS) scores ≥ 4, is not a rare complication after PKP, with an incidence ranging from 7.4% to 42.1% [5–7].

Rib fracture, subsequent new vertebral fracture, osteoporotic ostalgia, and other factors were reported to be associated with residual pain after PKP or PVP [5, 8]. Increasing studies have shown that preoperative thoracolumbar fascia injury (FI) is the most important risk factor for postoperative residual pain [6, 7, 9]. Thoracolumbar FI is detected based on MR findings of fascia edema, which can be manifested as short‐segment (≤ three levels) or long‐segment (> three levels) [7]. Previous studies have shown that the extent of the edema signal is positively correlated with the degree of postoperative residual pain, and the existence of FI significantly has a negative effect on the therapeutic efficacy of PKP or PVP [7, 10].

Although FI has received increasing attention, there is currently no targeted treatment for FI [9]. The postoperative residual pain caused by FI prevents patients from early ambulation and affects patients’ satisfaction with surgery, especially at the early postoperative stage. Therefore, relieving residual pain caused by FI is particularly important, as it would further improve the efficacy of minimally invasive vertebral augmentation surgeries and make patients more confident in early ambulation. Local injection therapy can significantly relieve pain caused by local inflammation or soft tissue injuries and obtain lasting pain relief [11]. The literature has reported that local injection therapy presented favorable efficacy in relieving pain caused by lumbar muscle strain, myofascitis, or osteoarthritis [12, 13]. However, whether local injection therapy is valid in treating FI caused by OVF and decreasing the incidence of postoperative residual pain is unknown. The purpose of this study was to evaluate the efficacy of PKP combined with local injection therapy for the treatment of OVF patients with FI.

The current study represents a continuation of our previous work and is part of a series of investigations on FI and residual pain after kyphoplasty. In our earlier study, we focused on identifying the significance of FI in residual pain and demonstrated how different severities of FI influenced postoperative outcomes [7]. The present study builds upon those findings and shifts the emphasis from observation to intervention. Specifically, it investigates the role of pre‐emptive local injection therapy targeted at FI, with the aim of alleviating early residual pain after kyphoplasty and ultimately providing a valuable reference for future clinical practice.

## 2. Materials and Methods

This study was approved by the Institutional Ethics Committee of the First Affiliated Hospital of Soochow University (No. 2023416).

### 2.1. Patient Population

Between January 2021 and December 2022, we enrolled 71 patients with OVFs who underwent PKP at our institution.

Inclusion criteria are as follows: (1) bone marrow edema of the index vertebra with posterior fascial injury on magnetic resonance imaging (MRI) and (2) single‐level OVF at T11–L2; (3) refractory back pain for ≥ 1 week despite conservative therapy.

Exclusion criteria are as follows: (1) pathological fractures due to spinal infection or metastatic tumor; (2) severe cardiac disease or hepatic/renal failure precluding anesthesia or surgery; (3) severe fractures with intervertebral dislocation or neurological deficit; (4) inability to complete data collection independently; and (5) other conditions that could confound pain assessment, including obvious lumbar spinal stenosis, lumbar disc herniation, a history of vertebral or rib fracture, and any other potentially confounding painful disorder.

In this study, 39 patients who underwent PKP alone were classified into Group 1, while 32 patients who were treated by PKP and local injection therapy were classified into Group 2. Moreover, the patients in Group 2 were further classified into two groups according to the severity of FI [7]. 15 patients with severe FI (> three levels) were grouped into the severe FI Group, and 17 patients with nonsevere FI (≤ three levels) were grouped into the nonsevere FI Group (Figure 1).

**FIGURE 1:**
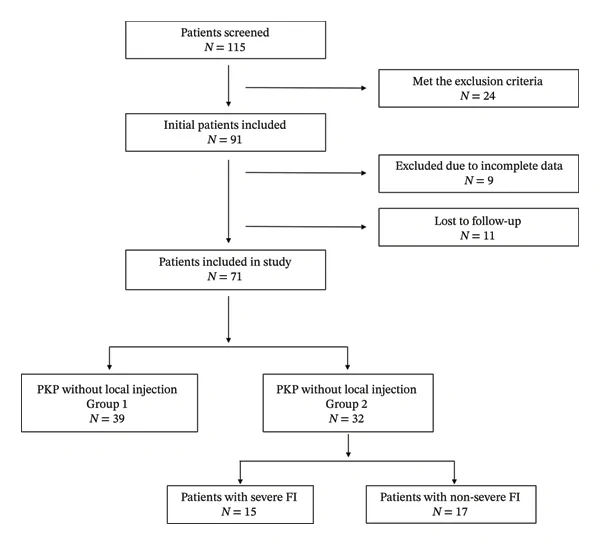
Flowchart of patients included and excluded in this study.

### 2.2. Surgical Procedure

PKP procedure: PKP was performed under general anesthesia with the patient in the prone position. After balloon‐mediated fracture reduction, polymethylmethacrylate (PMMA) cement was injected into the vertebral body under continuous fluoroscopic guidance to stabilize the fracture. The total cement volume was recorded.

Local injection therapy procedure: A mixed suspension of 0.5% lidocaine and compound betamethasone was prepared before the operation. Generally, 10 mL of the mixed suspension was prepared with a specific formulation of 2.5 mL of 2% lidocaine, 1 mL of compound betamethasone (containing betamethasone dipropionate 5 mg and betamethasone sodium phosphate 2 mg), and 6.5 mL of 0.9% sodium chloride injection. The injection volume depended on the length of the fascial injury on preoperative MRI [7]. Typically, a total of 2 mL was injected per level across both sides; if a larger volume was required, the same proportions were used. Taking the L1 level as an example, the target level was localized under fluoroscopy. Specifically, the injection sites were located approximately 1–3 cm lateral to the spinous process, between the mid‐level of the L1 pedicle shadow and the midpoint of the line connecting the L1 and L2 pedicle shadows. The skin and subcutaneous tissues were infiltrated in layers, and the needle tip was advanced under fluoroscopy into the area corresponding to the fascial injury on the preoperative MRI. The mixed suspension was then injected, and gentle local pressure was applied to promote diffusion. Early mobilization was initiated on postoperative Day 1, and all patients received standard antiosteoporosis therapy.

### 2.3. Clinical and Radiological Evaluation

Patients were followed in the outpatient clinic, and outcomes were recorded preoperatively, at postoperative Day 1, month 1, month 3, and at the final visit. Preoperative data included bone mineral density (BMD), body mass index (BMI), and the morphological pattern of the fractured vertebra (wedge, biconcave, or crush). Furthermore, the severity of fractured vertebrae was evaluated according to a previous study [7].

Pain was assessed at each time point using a 0–10 VAS (VAS; 0 = no pain, 10 = worst imaginable pain) score. Functional status was evaluated using the ODI score, calculated with the sexual function item excluded. Adverse events related to local injection were recorded after surgery and during follow‐up.

In addition, all patients were evaluated on their satisfaction with the efficacy of kyphoplasty one day after surgery [14]. Early patient satisfaction was captured using a study‐specific single‐item five‐level Likert question on postoperative Day 1. The result was classified into five degrees (very or somewhat satisfied, neutral, somewhat, or very dissatisfied).

### 2.4. Statistical Analysis

Statistical analysis was conducted using SPSS 19.0 software (SPSS Inc, Illinois, USA). Continuous variables were presented as mean ± standard deviation. Continuous variables were presented as mean ± standard deviation. The differences between the two groups were compared by the *t*‐test or Mann–Whitney *U* test for continuous variables and Chi‐square test for categorical variables. Paired *t*‐test or Wilcoxon signed‐rank test was used to compare the differences at different follow‐up times. A *p* value of less than 0.05 was considered to be statistically significant. Bonferroni correction was applied separately to the four postoperative comparisons for VAS and ODI between the two groups, giving a two‐sided significance threshold of *p* < 0.0125 (0.05/4) for each outcome. For the satisfaction survey, which was assessed only once on postoperative Day 1, a single comparison was performed using the Mann–Whitney *U* test, and a two‐sided threshold of *p* < 0.05 was considered statistically significant.

## 3. Results

There were 61 female and 10 male patients with an average age of 72.90 ± 7.54 years (range, 58–90 years) who were included in this study (Figure 2). The patients were followed up for a mean of 12.39 ± 1.33 months (range, 10–15 months; 12.21 ± 1.34 months in Group 1 and 12.63 ± 1.29 months in Group 2, respectively). The detailed demographic data of all the included patients are listed in Table 1. All the surgeries were performed successfully without severe complications. No injection‐related adverse events, such as local infection, hematoma, allergic reaction, neurological injury, or steroid‐related complications, occurred. The operative time was significantly longer in Group 2 than in Group 1 (50.97 ± 8.40 vs. 41.74 ± 6.09, *p* < 0.001). No differences were detected in demographic data between the patients with and without local injection.

**FIGURE 2:**
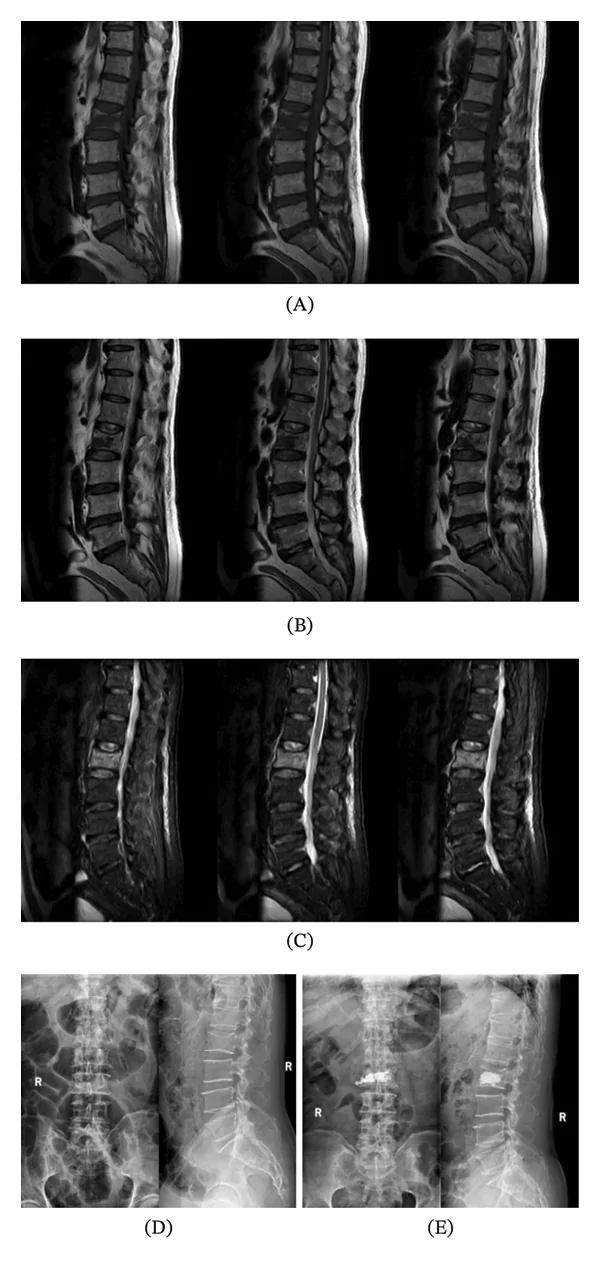
MR and X‐ray findings of L2 vertebral fracture before and after kyphoplasty. A 76‐year‐old male patient with back pain for 7 days. The T1‐weighted, T2‐weighted, and T2‐weighted fat suppression MR images (A–C) show an L2 vertebral fracture with severe FI. Preoperative anteroposterior and lateral X‐ray images (D) show L2 vertebral compression. Postoperative anteroposterior and lateral X‐ray images (E) present satisfactory cement distribution after percutaneous kyphoplasty surgery.

**TABLE 1:** Demographic data of patients treated by kyphoplasty with and without local injection therapy.

	Group 1 (*n* = 39)	Group 2 (*n* = 32)	*p* value
Age (year)	74.05 ± 7.63	71.50 ± 7.30	0.16
Gender (female/male)	34/5	27/5	0.74
BMD (T score)	−3.07 ± 0.32	−2.96 ± 0.29	0.15
BMI (kg/m^2^)	24.13 ± 3.32	22.95 ± 3.30	0.14
Fracture chronicity (day)	18.18 ± 6.71	16.03 ± 6.74	0.18
Preoperative analgesic use (yes/no)	8/31	10/22	0.30
Fracture level			0.26
T11	5	2	
T12	18	15	
L1	14	9	
L2	2	6	
Operative time (minute)	41.74 ± 6.09	50.97 ± 8.40	< 0.001^∗^
Fracture type			0.48
Wedge	26	19	
Biconcave	13	12	
Crush	0	1	
Fracture severity			0.73
< 25%	12	8	
25%–50%	22	21	
51%–75%	4	3	
> 75%	1	0	
Total volume of injected cement (mL)	7.15 ± 1.07	7.26 ± 0.93	0.39
Follow‐up time (month)	12.21 ± 1.34	12.63 ± 1.29	0.16

*Note:* Group 1 received PKP alone; Group 2 received PKP combined with local injection therapy.

Abbreviations: BMD = bone mineral density, BMI = body mass index.

^∗^Statistical significance was defined as *p* < 0.05.

As shown in Table 2, the VAS scores significantly decreased after surgery in both Group 1 and Group 2. However, the VAS scores were higher one day (3.67 ± 0.84) and 1 month (3.28 ± 0.83) after surgery in those patients without local injection therapy than those with injection therapy (2.72 ± 0.46 one day after surgery; 2.63 ± 0.61 1 month after surgery) (*p* < 0.001). At 3 months, the VAS scores showed no statistically significant difference between the two groups (2.46 ± 0.60 in Group 1 and 2.28 ± 0.52 in Group 2; *p* > 0.05).

**TABLE 2:** The VAS and ODI scores of OVF patients treated with or without local injection therapy evaluated before and after surgery.

		Group 1 (*n* = 39)	Group 2 (*n* = 32)	*p* value
VAS	preoperative	7.41 ± 0.75	7.66 ± 0.70	0.19
	one day postoperative	3.67 ± 0.84	2.72 ± 0.46	< 0.001^∗^
	1 month postoperative	3.28 ± 0.83	2.63 ± 0.61	< 0.001^∗^
	3 months postoperative	2.46 ± 0.60	2.28 ± 0.52	0.14
	last follow‐up	2.33 ± 0.58	2.13 ± 0.55	0.16

ODI	preoperative	66.23 ± 5.28	67.44 ± 5.26	0.34
	one day postoperative	37.44 ± 9.99	28.34 ± 5.60	< 0.001^∗^
	1 month postoperative	35.54 ± 8.93	29.38 ± 6.50	0.001^∗^
	3 months postoperative	22.38 ± 3.31	21.53 ± 3.39	0.30
	last follow‐up	22.79 ± 3.21	21.84 ± 3.19	0.19

*Note:* Group 1 received PKP alone; Group 2 received PKP combined with local injection therapy.

Abbreviations: ODI = Oswestry disability index, VAS = visual analogue scale.

^∗^Bonferroni correction was applied across the four postoperative between‐group comparisons within each outcome; *p* < 0.0125 was considered statistically significant.

The ODI scores presented similar trends to VAS scores. The values in Group 1 were 37.44 ± 9.99 one day after surgery and 35.54 ± 8.93 1 month after surgery which were significantly different from 28.34 ± 5.60 one day after surgery and 29.38 ± 6.50 1 month after surgery in Group 2, respectively (*p* < 0.001 on day one and *p* = 0.001 at 1 month). No statistically significant differences were detected 3 months after surgery and at the last follow‐up between the two groups (Table 2). The satisfaction survey one day after surgery showed that more patients expressed satisfaction with surgery (93.75%) in Group 2 compared to Group 1 (69.23%) (Table 3).

**TABLE 3:** The results of satisfaction survey of patients treated with or without local injection therapy evaluated one day after surgery.

	Group 1 (*n* = 39)	Group 2 (*n* = 32)	*p* value
Satisfaction of surgery			0.007^∗^
Very satisfied	19	25	
Somewhat satisfied	8	5	
Neutral	9	1	
Somewhat dissatisfied	3	1	
Very dissatisfied	0	0	

*Note:* Group 1 received PKP alone; Group 2 received PKP combined with local injection therapy.

^∗^Mann–Whitney *U* test was used for this ordinal data. Bonferroni correction was not applied here because satisfaction was assessed at a single time point. A *p* value < 0.05 was considered statistically significant.

Furthermore, the evaluation of the efficacy of PKP combined with local injection therapy for patients with severe or nonsevere FI showed that there were no statistically significant differences in VAS and ODI scores at each follow‐up time point (Table 4). The satisfaction survey also indicated that there was no significant difference between the two groups one day after surgery (Table 5).

**TABLE 4:** The VAS and ODI scores of OVF patients with severe or nonsevere fascia injury treated by local injection therapy evaluated before and after surgery.

		Severe FI group (*n* = 15)	Non‐severe FI group (*n* = 17)	*p* value
VAS	preoperative	7.47 ± 0.64	7.82 ± 0.73	0.19
	One day postoperative	2.67 ± 0.49	2.76 ± 0.44	0.66
	One month postoperative	2.53 ± 0.64	2.71 ± 0.59	0.43
	Three months postoperative	2.13 ± 0.52	2.41 ± 0.51	0.23
	Last follow‐up	2.00 ± 0.54	2.24 ± 0.56	0.33

ODI	preoperative	67.27 ± 4.57	67.59 ± 5.94	0.97
	One day postoperative	29.33 ± 6.99	27.47 ± 4.03	0.36
	One month postoperative	29.87 ± 5.96	28.94 ± 7.10	0.97
	Three months postoperative	20.93 ± 2.96	22.06 ± 3.73	0.91
	Last follow‐up	20.80 ± 2.96	22.76 ± 3.19	0.15

*Note:* Bonferroni correction was applied across the four postoperative between‐group comparisons within each outcome; p < 0.0125 was considered statistically significant

Abbreviations: ODI = Oswestry disability index, VAS = visual analogue scale.

**TABLE 5:** The results of satisfaction survey of patients with severe or nonsevere fascia injury treated by local injection therapy evaluated one day after surgery.

	Severe FI group (*n* = 15)	Non‐severe FI group (*n* = 17)	*p* value
Satisfaction of surgery			0.072
Very satisfied	14	11	
Somewhat satisfied	1	4	
Neutral	0	1	
Somewhat dissatisfied	0	1	
Very dissatisfied	0	0	

*Note:* Mann–Whitney *U* test was used for this ordinal data. Bonferroni correction was not applied here because satisfaction was assessed at a single time point. A *p* value < 0.05 was considered statistically significant.

## 4. Discussion

With the increasing application of PKP in treating OVFs, postoperative complications and related problems have attracted increasing attention [15, 16]. Postoperative residual pain, especially at the early stage, irritates patients, causing anxiety and depression, and even affecting surgical expectations. Residual pain after PKP surgery is not rare; the incidence of residual pain reported in the literature ranged from 7.4% to 42.1% [5–7]. Li et al. demonstrated that independent risk factors for residual back pain after PKP include the presence of an intravertebral vacuum cleft, posterior FI, facet joint violations, and a separated cement distribution [5]. Additionally, Yang et al. considered that preoperative low BMD, FI, multiple‐segment PVP, insufficient injected cement volume, unsatisfactory cement distribution, and depression were strong risk factors associated with residual pain after PKP in OVF patients [10]. Yan et al. reported that there was a significant relationship between FI and residual back pain after PVP [6].

OVF patients with FI present higher VAS and ODI scores after PKP, which affects the efficacy of PKP surgery [6, 7, 9, 10]. A prospective cohort study by Yan et al. showed that OVF patients with FI have relatively higher VAS and ODI scores [6]. Yang et al. reported that immediate postoperative VAS scores for OVF with FI are remarkably higher than those of OVF without FI after surgery [10]. Deng et al. found that the presence of FI significantly affected the effectiveness of PKP in treating OVF, resulting in worse VAS and ODI scores after PKP, and VAS score was positively correlated with the severity of FI [7]. The larger the extent of FI, the higher the VAS scores [7]. However, at present, there is still no study targeting how to treat OVF patients with FI.

Local pain caused by soft tissue injury or inflammation can be relieved effectively by local injection therapy, which is also widely used in the conservative treatment of osteoarthritis [13, 17, 18]. Ueshima et al. suggested that bilateral thoracolumbar interfascial plane block (TLIPB) combined with general anesthesia provides more effective perioperative pain relief than general anesthesia alone in patients undergoing spinal surgery and thus enhances their confidence in functional exercises soon after surgery [19]. Moreover, local injection therapy has also been reported in PKP surgery in several studies. Lin et al. reported that local anesthetic injection combined with PKP can reduce the intensity of early postoperative distal lumbosacral pain in patients with OVF [12]. Tao et al. performed TLIPB for patients who underwent PKP and found that the intra‐ and postoperative pain scores in the local injection group were significantly lower compared with the control group, indicating that local injection therapy can provide effective analgesia for back pain [20]. The mechanism of the analgesic effect of local injection in these studies may be that dorsal branches of spinal nerves innervate paraspinal muscles and retrospinal bone elements, and blocking the dorsal ramus of the nerve which passes through paraspinal tissue, can provide effective analgesia for back pain.

Regional anesthesia techniques, such as the TLIP block, are generally performed under ultrasound guidance by injecting local anesthetic into the anatomical plane between the multifidus and longissimus muscles to block the dorsal rami. In contrast, the procedure used in this study was MRI‐targeted local infiltration rather than a conventional interfascial plane block. The injection level was selected according to the area of most pronounced thoracolumbar fascia edema at the fractured vertebra, and a mixture of lidocaine, betamethasone, and sodium chloride was injected bilaterally.

In this study, local fascia injection therapy was performed to evaluate the efficacy of pain relief in OVF patients with FI. According to the extent of FI detected by MRI, we performed local injection after PKP surgery, and satisfactory results were obtained. The VAS and ODI scores one day and 1 month after surgery in the local injection group were significantly better than those in the control group. Within the injection group, no statistically significant differences in VAS or ODI scores were found between the severe and nonsevere FI subgroups at the assessed time points, indicating that local fascia injection can achieve satisfactory results regardless of the severity of FI [7]. The mechanism of residual pain caused by FI is mainly considered to be the stimulation of the peripheral spinal nerve branches by thoracolumbar FI, and local injection can block these nerves, which leads to satisfactory pain relief at an early stage. These results indicated that PKP surgery combined with local injection therapy significantly reduced the incidence of early postoperative residual back pain. The satisfaction survey also presented that more patients expressed their satisfaction with surgery when local injection was also performed for those OVF patients with FI. Thus, fascia local injection may therefore be considered for early symptom relief, although the operation time of the local injection group was significantly longer than that of the control group.

Our study only included patients with single‐level OVF, so the findings may not directly apply to those with fractures at multiple levels. In multilevel OVFs, fascial injury may be more extensive, and the sources of pain are more complex than in the single‐level OVF patients included in this study. Evaluating the efficacy of local injection for multilevel OVF with fascial injury is therefore difficult and subject to confounding. Studies focusing on multilevel fractures are still needed to assess the efficacy and safety of local injection in this group.

This study has several limitations. First, it is retrospective and nonrandomized. Treatment allocation was based on shared decision‐making between patients and treating surgeons rather than randomization; therefore, selection bias and confounding by indication cannot be excluded. Second, in this retrospective study, the frequency and intensity of preoperative analgesic and antiosteoporosis medication use could not be uniformly recorded or quantified in the two groups. This may have affected patients’ pain scores and is a limitation of our study. The satisfaction outcome used a study‐specific single‐item measure without formal psychometric validation; thus, its findings are interpreted cautiously. Studies with higher levels of evidence and more cases are needed to further explore the clinical significance of local injection combined with PKP in OVF patients with FI.

## 5. Conclusions

Local injection therapy combined with PKP is a safe and effective strategy for improving early postoperative recovery and reducing early residual pain in OVF patients with FI. Therefore, this approach could prove to be effective in clinical practice and needs to be further investigated.

## Author Contributions

Junjie Niu, Jinning Wang, and Jun Zou contributed to the study conception and design. Data collection and analysis were performed by Zicheng Deng, Luyang Li, Tao Feng, Yufan Ying, Dawei Song, Shengyang Jin, and Qi Yan. The first draft of the manuscript was written by Zicheng Deng, Luyang Li, Tao Feng, and Jinning Wang. Junjie Niu, Dawei Song, and Jun Zou revised previous versions of the manuscript.

## Funding

No funding was received for this manuscript.

## Disclosure

All authors read and approved the final manuscript.

## Conflicts of Interest

The authors declare no conflicts of interest.

## Data Availability

The data that support the findings of this study are available from the corresponding authors upon reasonable request.
